# Dengue Virus in Sub-tropical Northern and Central Viet Nam: Population Immunity and Climate Shape Patterns of Viral Invasion and Maintenance

**DOI:** 10.1371/journal.pntd.0002581

**Published:** 2013-12-05

**Authors:** Maia A. Rabaa, Cameron P. Simmons, Annette Fox, Mai Quynh Le, Thuy Thi Thu Nguyen, Hai Yen Le, Robert V. Gibbons, Xuyen Thanh Nguyen, Edward C. Holmes, John G. Aaskov

**Affiliations:** 1 Centre for Immunity, Infection and Evolution, University of Edinburgh, Edinburgh, United Kingdom; 2 Oxford University Clinical Research Unit, Hospital for Tropical Diseases, Ho Chi Minh City, Vietnam; 3 Centre for Tropical Medicine, Nuffield Department of Clinical Medicine, University of Oxford, Oxford, United Kingdom; 4 Oxford University Clinical Research Unit, Wellcome Trust Major Overseas Programme, Hanoi, Vietnam; 5 National Institute of Hygiene and Epidemiology, Hanoi, Vietnam; 6 Military Institute of Hygiene and Epidemiology, Hanoi, Vietnam; 7 Armed Forces Research Institute of Medical Sciences, Bangkok, Thailand; 8 Marie Bashir Institute for Emerging Diseases and Biosecurity, School of Biological Sciences and Sydney Medical School, The University of Sydney, Sydney, New South Wales, Australia; 9 Fogarty International Center, National Institutes of Health, Bethesda, Maryland, United States of America; 10 Queensland University of Technology, Brisbane, Australia; 11 Australian Army Malaria Institute, Brisbane, Australia; University of California, Davis, United States of America

## Abstract

Dengue virus transmission occurs in both epidemic and endemic cycles across tropical and sub-tropical regions of the world. Incidence is particularly high in much of Southeast Asia, where hyperendemic transmission plagues both urban and rural populations. However, endemicity has not been established in some areas with climates that may not support year-round viral transmission. An understanding of how dengue viruses (DENV) enter these environments and whether the viruses persist in inapparent local transmission cycles is central to understanding how dengue emerges in areas at the margins of endemic transmission. Dengue is highly endemic in tropical southern Vietnam, while increasingly large seasonal epidemics have occurred in northern Viet Nam over the last decade. We have investigated the spread of DENV-1 throughout Vietnam to determine the routes by which the virus enters northern and central regions of the country. Phylogeographic analysis of 1,765 envelope (E) gene sequences from Southeast Asia revealed frequent movement of DENV between neighboring human populations and strong local clustering of viral lineages. Long-distance migration of DENV between human population centers also occurred regularly and on short time-scales, indicating human-mediated viral invasion into northern Vietnam. Human populations in southern Vietnam were found to be the primary source of DENV circulating throughout the country, while central and northern Vietnam acted as sink populations, likely due to reduced connectedness to other populations in the case of the central regions and to the influence of temperature variability on DENV replication and vector survival and competence in the north. Finally, phylogeographic analyses suggested that viral movement follows a gravity model and indicates that population immunity and physical and economic connections between populations may play important roles in shaping patterns of DENV transmission.

## Author Summary

Reports from sub-tropical regions of the world suggest a growing risk of introduction and establishment of dengue viruses (DENV) in new locales. Recent dengue epidemics in northern Viet Nam present an opportunity to study how DENV invades and spreads in these environments. The proximity of this region to tropical areas experiencing year-round endemic DENV transmission makes it an ideal site for studying the effects of human population movement and climate on DENV emergence. We performed a phylogenetic analysis using DENV-1 envelope gene sequences from Southeast Asia. We show that DENV are regularly imported into northern and central Viet Nam from southern Vietnam, and that increasingly large seasonal epidemics in the north are caused by newly introduced viruses each year. While tropical Vietnam maintains localized virus populations for multiple years, cool winter temperatures in sub-tropical northern Viet Nam may reduce mosquito populations and virus replication to levels that are not conducive to year-round DENV transmission. Finally, we found that the dispersal of DENV across the region is well-described using human movement and immunity data, and believe that increased epidemiological, entomological, and virological surveillance are needed to understand the processes by which endemic DENV transmission becomes established in new populations.

## Introduction

Dengue viruses (DENV) are single-stranded, positive-sense, mosquito-borne RNA viruses (family *Flaviviridae*), within which considerable genetic diversity is present on both global and local scales [1]. All four serotypes (DENV-1 to DENV-4) are capable of infecting humans and result in a spectrum of clinical outcomes, ranging from asymptomatic to severe disease. In the 1960s, fewer than ten countries reported a total of ∼15,000 dengue cases to WHO annually [2], [3]. Rapid urbanization and global travel have fueled the global spread and establishment of DENV populations across the tropics and sub-tropics, and recent estimates suggest that approximately 96 million symptomatic cases now occur in more than 120 countries every year [4].

Endemic DENV transmission occurs throughout the tropics, while sub-tropical regions experience epidemics of varying size that subside with the shift to winter temperatures. Little to no detectable transmission occurs in these sub-tropical areas during cooler months and, in some areas, DENV transmission remains at very low levels for several years following an epidemic [5]–[7]. Phylogenetic studies of DENV from southern China and Taiwan suggest that seasonal epidemics often originate with DENV-infected travelers arriving from endemic areas of Southeast Asia [8]–[13]. Due to a lack of long-term virological surveillance in regions where dengue outbreaks are sporadic, it is not clear whether DENV lineages persist across seasons in these environments. In addition, there has been no investigation of the processes by which DENV are introduced and become established in human populations that are closely linked to endemic areas by land and air travel but sit at the geographic and climatic margins of endemic transmission. A better understanding of the invasion of DENV into these environments would provide important insights into the expansion of the geographic range of dengue and its emergence in sub-tropical environments.

Human populations in southern Viet Nam have supported hyperendemic DENV transmission since at least the early 1960s and now experience fairly stable endemic transmission [14]–[19]. In contrast, dengue is considered to be ‘emerging’ in northern Viet Nam, where the annual incidence has increased over the previous decade [5]. While the clinical burden of dengue across Southeast Asia is generally found in children less than 15 years of age, 85% of dengue cases reported in Hanoi (the capital of Viet Nam, located in the north of the country) occur in adults [20]. This suggests later exposure to DENV, reduced population immunity, and thus a lower force of infection or a lack of hyperendemic transmission in northern Viet Nam relative to southern Viet Nam [5], [21], [22]. Climate is likely a major factor in these geographical differences in incidence and transmission intensities, as cool winter temperatures in northern Viet Nam may reduce mosquito breeding and survival and increase extrinsic incubation times such that year-round endemic transmission is inhibited [23]–[26].

Revealing how DENV move between geographic localities is integral to understanding dengue epidemiology in both endemic and epidemic areas. While the molecular epidemiology of DENV has been investigated in southern Viet Nam and neighboring countries [17], [18], [27]–[30], the DENV populations of northern and central Viet Nam have not been described. Moreover, after a decade of increasing DENV activity, it is not clear whether year-round autochthonous transmission of local lineages of DENV occurs in northern Vietnam, or if viral populations die out there in the winter months and are reseeded from endemic areas (particularly central and southern Viet Nam) every year. Understanding the epidemiology and evolution of this emerging pathogen at the margins of transmission will provide valuable insights into the process by which DENV transitions from epidemic to endemic transmission, and may reveal factors that influence pathogen emergence in human populations.

The aim of this study was to investigate the spread of DENV-1 throughout Viet Nam over the course of a decade and to determine the routes by which viral populations enter northern and central Viet Nam. For this, we utilized a large data set (n = 1,765 sequences) of DENV-1 envelope gene sequences collected from Vietnamese hospitals and studies across Southeast Asia, where Genotype I has been the dominant circulating DENV-1 lineage since at least 1980. With these data, we investigated the movement of this lineage into Viet Nam and addressed the following questions: (i) Does highly endemic southern Viet Nam act as a source population for DENV circulating in other parts of the country? (ii) Do DENV populations persist over multiple seasons in central and northern Viet Nam? (iii) What factors determine the patterns of dispersal of DENV lineages to new environments across Viet Nam and within Southeast Asia?

## Materials and Methods

### Viral sampling and envelope gene sequencing

Dengue viruses were recovered from suspected dengue patients presenting to hospitals across Viet Nam as part of routine diagnostic serology. The envelope (E) genes of 60 isolates recovered from north and central Viet Nam and one from southern Viet Nam were sequenced as described previously [31], [32] and have been submitted to Genbank (submission numbers 1608348 and 1608374). Twenty additional DENV-1 viruses were collected from cases presenting to The National Hospital for Tropical Diseases in Hanoi and sequenced using standard Sanger sequencing methods. These have been assigned GenBank accession numbers HQ591537-HQ591556. The geographic and temporal distribution of all Vietnamese sequences is shown in Table S1.

### Phylogenetic analysis

DENV-1 E gene sequences from northern and central Viet Nam were combined with full-length DENV-1 E gene sequences catalogued in GenBank to comprise all DENV-1 sequences from across Asia for which the year and country of sampling were known. Nucleotide alignments of 1765 full-length DENV-1 E gene sequences (1485 nt), including the 80 isolates from northern and central Viet Nam, were manually constructed using Se-AL [33]. To infer phylogenetic relationships for the complete data set of DENV-1 sequences and identify geographic regions with phylogenetic links to northern and central Viet Nam, we utilized the maximum likelihood (ML) method available in PhyML, incorporating a GTR model of nucleotide substitution with gamma-distributed rate variation among sites and a heuristic SPR branch-swapping search algorithm [34]. This initial analysis indicated that all northern and central Vietnamese DENV-1 sequences belong to a Southeast Asian subset of Genotype I, comprising viral sequences from Thailand, Cambodia, and southern Viet Nam, as well as a maritime Southeast Asian lineage based in Singapore, Malaysia, and Indonesia. Within the maritime Southeast Asian lineage, these isolates were most closely related to Singaporean viruses. A second alignment was then constructed using 80 DENV-1 E gene sequences from northern and central Viet Nam and 625 unique E gene sequences of viruses isolated from the surrounding regions between 1997 and 2009 and for which the exact date of sampling was known (Cambodia, Thailand, Singapore, southern Viet Nam). Small numbers of sequences and a lack of exact sampling dates for viral sequences from other countries in maritime Southeast Asia prevented us from including sequences from these countries in the analysis. Thus, viral, geographic, and epidemiological data from Singapore were used to represent the maritime Southeast Asian clade in all analyses. Phylogenetic analyses were undertaken using the Bayesian Markov Chain Monte Carlo (MCMC) method implemented in BEAST (v1.6.2), incorporating the date of sampling [35] and utilizing a codon-structured SDR06 model of substitution, a relaxed molecular clock as in [18], and a Bayesian skyline prior (BSP; 5 piecewise constant groups). The MCMC chain was run for 100 million iterations, with sub-sampling every 10,000 iterations. All parameters reached convergence as assessed visually using Tracer (v.1.5). The initial 10% of the chain was removed as burn-in, and maximum clade credibility (MCC) trees were summarized using TreeAnnotator (v.1.6.2).

### Spatial analysis

To investigate the routes of invasion of DENV-1 into northern and central Viet Nam, the geographic areas of Viet Nam were categorized using (i) a ‘Local geographic model’ – categorized by government-defined regions (MKD: Mekong Delta, HCM: Ho Chi Minh City, SE: Southeast, CHL: Central Highlands, SCC: South Central Coast, NCC: North Central Coast, RRD: Red River Delta), and by country outside of Viet Nam (KH: Cambodia, SG: Singapore, TH: Thailand), and (ii) a ‘Regional geographic model’ – categorized by larger regions (North: RRD and NCC, Central: CHL and SCC, South: SE, HCM and MKD) and elsewhere by country as in the previous scheme. We inferred rates of viral migration between locations using an asymmetric model of discrete diffusion across Southeast Asia and within Viet Nam [36]. Posterior distributions of trees were estimated under a phylogenetic model using the MCMC method implemented in BEAST (v1.6.2) using BEAGLE [35], [37]. This model incorporated the date of sampling and a relaxed molecular clock, Bayesian skyline prior, and the SRD06 codon position model as described above. The MCMC chain was run for 100 million iterations, with sub-sampling every 10,000 iterations, and all parameters reached convergence. The initial 10% of the chain was removed as burn-in, and Maximum Clade Credibility (MCC) trees including ancestral location-state reconstructions were summarized using TreeAnnotator (v.1.6.2). The expected number of location state transitions conditional on the location-related sequence data was determined using Markov Jump counts, summarized per branch and for the complete evolutionary history. Markov Jump counts of the expected number of geographic state transitions along branches provide a quantitative measure of gene flow between regions, representing successful viral introduction from one region to another, and are not heavily influenced by single isolate introductions [38], [39]. Finally, parsimony score (PS) and association index (AI) tests were utilized to assess the extent of geographic structure across all trees using the Bayesian Tip-association Significance Testing (BaTS) program [40] based on the posterior distribution of trees generated in the BEAST analysis described above.

To account for potential sampling biases in space and time, posterior distributions were also estimated as above for ten data sets that were subsampled randomly, with replacement, to include no more than 50 sequences per region (338 sequences total) for each of the ten major geographic regions in the data set (Cambodia, Singapore, Thailand, and Viet Nam: Mekong Delta, Ho Chi Minh City, Southeast, Central Highlands, South Central Coast, North Central Coast, Red River Delta) and for ten data sets that were subsampled such that no more than five samples per year were chosen randomly from each of the locations indicated above (205 sequences). In addition, six major clades containing northern and central Vietnamese isolates were identified in trees inferred from the full data set and were analyzed individually using the asymmetric discrete diffusion model to assess potential differences in the spatial and temporal patterns of diffusion among them, to identify routes of invasion into northern and central Viet Nam, and to determine whether lineages were maintained over multiple years in these newly sampled populations.

### Hypothesis-based spatial analysis

To test hypotheses related to viral dispersal and establishment, we set rate priors to specific values to construct a series of (asymmetric) phylogeographic models that might reflect the epidemiology and dispersal of DENV in Viet Nam. These models were analyzed using both the full and subsampled data sets for the Regional and Local geographic models. These models were: (i) a geographic diffusion model that assumes equal rates of viral migration between all regions of interest (Model 1, Equal Rates), (ii) a model based on the physical distance separating the populations in question using the inverse of the Euclidian distance between the centroids of the largest cities in each region (Model 2, Distance), (iii) a population size-based model, utilizing the census population estimate of the largest city from which sequences were collected in each region as representative of the influence of that region in attracting migration from other locations (Model 3, Population) [41]–[44], and (iv) a previously described gravity model incorporating geographic distances (calculated as in Model 2) and population size data from both the recipient and donor locations, in which distance and population size act as repelling and attracting forces, respectively (Model 4: Gravity Model) [17], [45]. Prior human immunity to DENV is likely to play an important role in the ability of viruses to invade populations [46]. Similarly, transmission intensity, as reflected in the average/median age of infection [21], will vary with time and hence influence patterns of spatial spread. To incorporate these factors into our phylogeographic models, we determined the ratio of the mean age of reported dengue cases in the recipient population to that in the donor population, and utilized this as a crude measure of the likelihood of viral invasion [5], [22], [47]–[51]. We refer to this ratio as the Relative Endemicity factor (REf) (Model 5, REf). Due to a lack of age-specific case data from local populations, Regional estimates were extrapolated to all locations within the same geographic region for the Local model. In Model 6, we integrated this immunity measure as a proportionality constant in gravity model calculations (REf+Gravity Model). Finally, we investigated the effects of sample size on phylogeographic inference using a rate matrix based on the sample size of the donor population (Model 7, Sample Size).

Model priors were normalized (mean one and unit variance) and incorporated into asymmetric matrices that allow for directional rates to vary between individual location pairs. A posterior simulation-based analogue Akaike's information criterion through MCMC (AICM) was implemented using likelihoods specific to the geographic model priors, and marginal log likelihood estimates for each model were compared to determine the best fit model to the data in hand [52], [53].

### Ethical approval

Sequencing of de-identified viruses collected in this study was undertaken under Human Research Ethics Approval 0700000910 from the Queensland University of Technology. Viruses collected at The National Hospital of Tropical Diseases in Hanoi, Viet Nam were from patients enrolled in a study approved by the scientific and ethical committees at the National Hospital of Tropical Diseases and The Oxford University Tropical Research Ethics Committee

(OXTREC) [54]. Patients provided written informed consent to participate in this study.

## Results

### Phylogeography of DENV-1 in Southeast Asia and Viet Nam

We determined the E gene sequences of DENV-1 isolates collected from 81 Vietnamese dengue patients and combined these with Southeast Asian DENV-1 E gene sequences collected between 1997 and 2009. The full data set included 46 sequences from northern Viet Nam (1998–2009), 34 sequences from central Viet Nam (2004–2009), and 461 sequences from southern Viet Nam (2003–2008), and 70, 63, and 31 envelope gene sequences from Thailand (1997–2007), Cambodia (2000–2008), and Singapore (2003–2008), respectively. All viruses were collected subsequent to a clade replacement event that occurred within the DENV-1 population in Thailand in the mid-1990s that has been attributed to enhanced transmission capacity within the vector [55]. This appears to have been the primary DENV-1 lineage circulating in mainland Southeast Asia for at least a decade, although the lack of samples from Cambodia and Viet Nam in early years prevents investigation of the means by which this lineage initially spread through the region.

**Figure 1:**
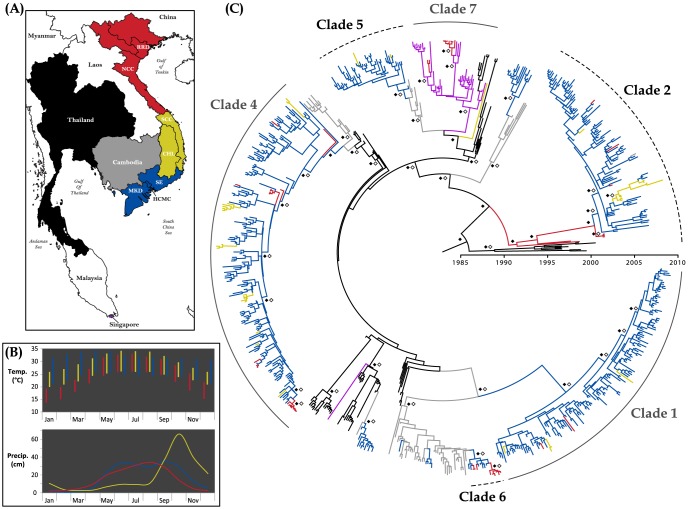
Regional phylogeography among 705 DENV-1 genotype 1 sequences isolated in Southeast Asia from 1998 to 2009. A) Map of Southeast Asia. B) Monthly averages of climate factors in the three primary regions of Viet Nam (mean maximum and mean minimum monthly temperatures, mean precipitation). Data for the largest city in each region (Hanoi, Danang, Ho Chi Minh City) were obtained from the World Meteorological Organization (http://worldweather.wmo.int/) and are colored as in A. C) Maximum clade credibility (MCC) tree showing phylogeographic relationships among Southeast Asian DENV-1 E gene sequences. Branch colors correspond to locations indicated in the map (Singapore shown in purple). Closed diamonds indicate posterior probability support ≥0.85. Open diamonds indicate ancestral location state probability ≥0.85.

Thailand, Cambodia, and Viet Nam all experienced the co-circulation and maintenance of multiple lineages for several years and importation of novel viruses from other countries (Figure 1). Our phylogeographic analyses provided no support for Cambodia or Viet Nam acting as a source of recent DENV-1 lineages circulating in Thailand [mean Markov Jump counts (95% highest posterior density [HPD]): KH to TH, 0.16 (0, 1); South VN to TH, 0.04 (0, 0); Central VN to TH, 0.08 (0, 1); North VN to TH, 0.54 (0, 3)], while moderate support was provided for migration routes from Thailand to Cambodia and from Cambodia to southern Viet Nam (Table 1, Table S2). Due to differences in sampling densities over time, conclusions on the geographic origins of some lineages cannot be made. However, lineages in which contemporaneous sequences are present in both Cambodia and Viet Nam (Clades 1, 4, 5, and 6) strongly support a Cambodian origin of Vietnamese DENV-1 populations (Figure 1). Although frequent movement of viruses between locations was observed between 1990 and 2009, very strong clustering by country and sub-national region within Viet Nam indicates that gene flow is much higher within the defined geographic areas than between them [Regional analysis – full phylogeny: Association Index, AI = 0.06 (0.05, 0.08), Parsimony Score, PS = 0.16 (0.15, 0.17), subsampled trees: averaged AI: 0.12 (0.10, 0.15), averaged PS = 0.25 (0.24, 0.27); Local analysis – full phylogeny: AI = 0.19 (0.17, 0.21), PS = 0.27 (0.25, 0.28), subsampled trees: averaged AI: 0.25 (0.22, 0.30), averaged PS = 0.38 (0.35, 0.40)].

**Table 1:** Viral migration patterns in the complete data set (full tree) and in Vietnamese clades.

Regional Model	TH to KH	SG to North VN	KH to South VN	South VN to Central VN	South VN to North VN	Within South VN (Local model only)
**Full tree**	4.9 (2, 7)	2.3 (1, 4)	8.7 (6, 11)	14.7 (13, 17)	14.4 (11, 17)	
**Clade 1**				3.0 (2, 3)	1.9 (1, 2)	
**Clade 2**				2.1 (1, 3)	3.6 (2, 6)	
**Clade 4**				7.4 (6, 9)	5.0 (3, 7)	
**Clade 5**				2.0 (1, 2)		
**Clade 6**					2.2 (1, 3)	
**Clade 7**		2.0 (1, 3)				

Local Model	TH to KH		KH to HCM	HCM to SCC	HCM to RRD	SE to RRD	HCM to SE	HCM to MKD	MKD to SE	MKD to HCM
**Full tree**	4.3 (1, 7)		6.9 (3, 10)	13.6 (10, 17)	6.0 (3, 9)	8.2 (4, 10)	12.3 (5, 18)	52.5 (43, 63)	11.1 (5, 16)	33.5 (21, 46)
**Clade 1**				2.8 (1, 4)			6.0 (2, 9)	4.5 (1, 8)	4.6 (1, 7)	22.1 (17, 27)
**Clade 2**								17.2 (13, 21)		
**Clade 4**				7.2 (5, 9)	4.5 (2, 6)		5.4 (2, 7)	20.1 (14, 25)		
**Clade 5**				1.9 (1, 2)				4.5 (3, 6)		
**Clade 6**										
**Clade 7**										

The number of viral introductions is represented by Markov Jump counts (posterior expected number of state transitions between location in Southeast Asia, with 95% highest posterior density (HPD) intervals). Only significant Markov Jump counts are shown.

A minimum of eight distinct lineages of DENV-1 Genotype 1 entered Viet Nam between 1990 and 2007 and persisted until at least 2007–2009. While all viral diversity captured within the country was represented in samples from the south, viral populations in the northern and central regions were less diverse. Nearly all viruses isolated from northern and central Viet Nam clustered within the diversity of the south with the exception of viruses isolated in northern Viet Nam prior to 2003 and a single divergent virus collected in the Central Highlands in 2004 (basal to Clades 5 and 7); notably, very few sequences were available from southern Viet Nam during this time period (nine in 2003, two in 2004). While strong support was found for a Cambodian origin of most Vietnamese lineages, Clades 7 and 2 may have been introduced from Singapore (or elsewhere in maritime Southeast Asia) and Thailand, respectively. Clade 7 contained two examples of importation of novel lineages of the maritime Southeast Asian clade into northern Viet Nam followed by localized, short-term transmission during a single year and apparent fade-out with the onset of winter (Table 2), when temperatures are low and vector populations are expected to be reduced. These analyses also suggested migration of viruses from maritime Southeast Asia into Thailand in 2004 followed by sustained co-circulation with indigenous Thai DENV-1 through the 2007 dengue season (Figure 1).

**Table 2:** Estimates of the Time to the Most Recent Common Ancestor (TMRCA) and the time of the last viral isolate of clusters circulating in central and northern Viet Nam.

Clade	Location	Mean TMRCA (95% HPD)	Most recent isolation date within cluster
**1**	**Central Viet Nam**	2008.9 (2008.6, 2009.0)	2009.4
**2**	**North Viet Nam**	1990.5 (1987.0, 1993.7)	2002.5
	**Central Viet Nam**	2003.9 (2003.4, 2004.2)	2009.5
**4**	**North Viet Nam**	2004.4 (2003.9, 2004.7)	2004.8
		2008.4 (2008.0, 2008.7)	2008.8
		2008.5 (2008.1, 2008.7)	2008.8
	**Central Viet Nam**	2006.6 (2006.3, 2006.9)	2007.3
		2008.5 (2008.0, 2008.8)	2009.5
		2008.5 (2008.1, 2008.9)	2009.3
		2008.8 (2008.3, 2009.0)	2009.6
**5**	**NA**		
**6**	**North Viet Nam**	2008.5 (2008.5, 2008.6)	2008.7
		2009.1 (2008.8, 2009.4)	2009.9
**7**	**North Viet Nam**	2006.0 (2005.9, 2006.3)	2006.5
		2008.1 (2007.7, 2008.6)	2008.9

A previous analysis of DENV-1 within southern Viet Nam indicated that Clade 2 became established there in 2002 [18]. The addition of isolates from northern Viet Nam in this study showed a considerably longer history of this lineage in the country, beginning in the late 1980s/early 1990s. Ancestral state reconstruction suggested that this lineage migrated from Thailand into northern Viet Nam, but Markov Jump counts at the basal node are quite low (Regional model: 0.18, Local model: 0.40) due to the existence of only a few sequences from viruses recovered during the early period of invasion and long branches between lineages. This was investigated further in clade-specific analyses and is discussed below.

### Viral migration within Viet Nam

Of six viral lineages involved in transmission within the central and northern regions of Viet Nam, five showed invasion and dispersal throughout the country and frequent movement between areas of interest. To investigate the spread of DENV-1 genotype 1 within Viet Nam, we estimated Markov Jump counts between locations across the full phylogeny and in subsampled data sets, and analyzed specific viral clades to investigate fine-scale spatial and temporal patterns of dispersal. Using the Regional asymmetric phylogeographic migration model, we determined that southern Viet Nam was the likely source for Vietnamese viruses in Clades 1, 4, 5, and 6 and for Clade 2 viruses isolated after 2002 (Table 1). No support was found for viral migration between the central and northern regions of the country [mean Markov Jump counts across the full phylogeny (95% HPD): central VN to northern VN, 0.23 (0, 1); northern VN to central VN, 0.26 (0, 1)], although the small numbers of sequences obtained from these regions may have obscured any such links if sampling was not representative of the full diversity in these locations. Finer scale spatial analysis using the Local model showed that DENV tended to move between neighboring areas, but also implicated Ho Chi Minh City as the primary source population for the entire country. Significant migration was detected from this densely populated urban area into the surrounding Mekong Delta and Southeast regions, as well as to the South Central Coast and the distant Red River Delta (Table 1, Figure S1). These relationships were consistent across most clades and within sub-sampled data sets (Table S2), which suggested that inferred relationships are not an artifact of dense sampling in the south.

**Figure 2:**
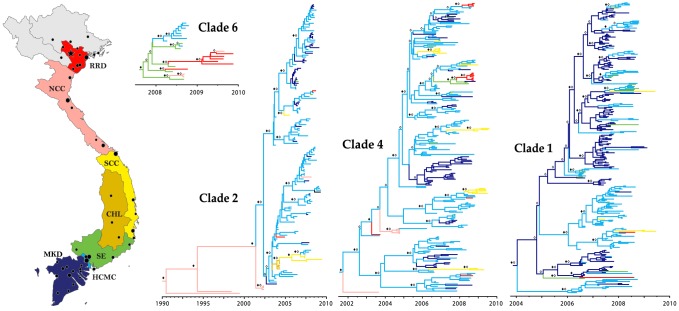
Local phylogeography in a sample of Vietnamese DENV-1 clades. Maximum clade credibility (MCC) trees showing phylogeographic relationships among Vietnamese DENV-1 E gene sequences. Branch colors correspond to locations indicated in the map of Viet Nam. Closed diamonds indicate posterior probability support ≥0.85 on relevant nodes. Open diamonds indicate ancestral location state probability ≥0.85. Black circles on the map indicate cities with population size greater than 100,000, with the size of the circle representing the relative population size. The black star indicates Viet Nam's capital city, Hanoi.

The Mekong Delta and Southeast regions also acted as secondary centers of viral diversity within the country. The Mekong Delta region is the inferred entry site of Clade 1 into Viet Nam. Long-term transmission of these viruses as well as sub-lineages in Clades 2 and 4 also occurred in the Mekong Delta. However, viruses only disseminated from this region into areas with which it shares borders, namely Ho Chi Minh City and the Southeast (Table 1, Figure S1). The Mekong Delta acted as a significant source for these populations across the full phylogeny based largely on several migration events and subsequent establishments in Ho Chi Minh City in Clade 1 (Table 1, Figure 2). Notably, no links were detected between the DENV populations in the Mekong Delta and central or northern viral populations. In contrast, the Southeast region did not appear to play a significant role in maintaining diversity within the south and instead generally acted as an acceptor of viruses from Ho Chi Minh City and, to a lesser extent, from the Mekong Delta. While limited sampling could have obscured a role for this region as a source population in the south, analysis of the full data set revealed that the area is a significant source of viral populations appearing in the Red River Delta (Table 1), and branch-specific Markov Jump counts suggest this area as an independent source of established DENV populations in the Red River Delta and North Central Coast in Clades 4 and 6 (Figure S1).

### Invasion and maintenance of DENV-1 in northern and central Viet Nam

To determine whether distinct viral populations persisted in northern and central Viet Nam over multiple seasons, we investigated the timing of invasion and establishment of novel viral sub-lineages (that is, viral clusters of two or more sequences originating from the same location as inferred by ancestral state reconstruction) and the potential co-circulation of distinct lineages in these areas. Viral invasion and establishment were detected in northern Viet Nam in 1990, 2004, 2008, and 2009, and in central Viet Nam in 2003, 2006, and 2008. Except in Clade 2, these data suggest that invading lineages in northern Viet Nam did not persist in the region over multiple dengue seasons (Table 2).

The times to most recent common ancestry (TMRCAs) of viral clusters in northern Viet Nam suggested that viruses are imported to the region throughout the year, although most invasion events occurred in the middle of the year via viral migration from the south (Ho Chi Minh City and the Southeast). This period coincides with seasonal increases in the number of dengue cases throughout the country and in Hanoi [5], when viral migration and establishment are more likely due to high levels of DENV transmission in the south and suitable climate conditions for the vector in the north.

In contrast to the north, DENV from six central Vietnamese transmission clusters (one each in 2003, 2006, and four clusters in 2008) suggested that seasonal invasion in this region occurred during the ‘dengue season’ in the second half of the year (Table 2), with uninterrupted transmission often maintained for multiple years. Among these persistent lineages, one central Vietnamese sub-lineage in Clade 2 became established in the Central Highlands around 2003 with viruses from this lineage later isolated in the South Central Coast region, where it was maintained into the 2009 dengue season. The concurrent invasion and co-circulation of multiple clades was common in the South Central Coast region (two in 2006, Clades 2 and 4; five in 2008–2009, Clades 1, 2 and 4).

### Testing models of DENV phylogeography

**Figure 3:**
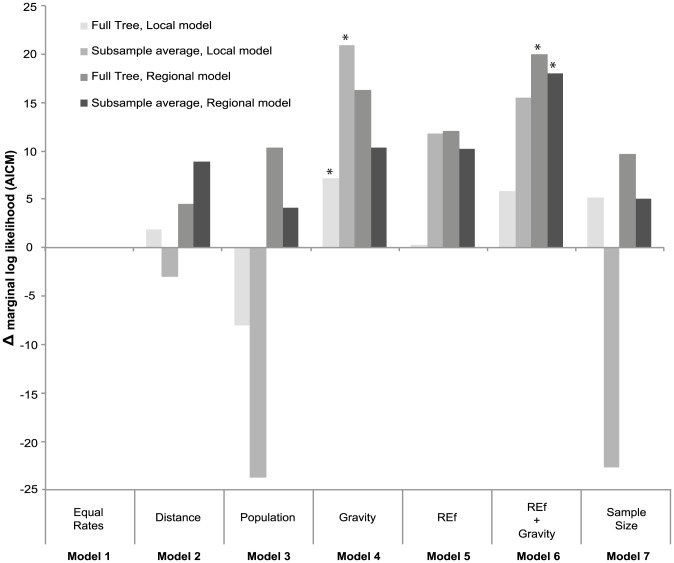
Differences in marginal log likelihood estimates for the fit of different phylogeographic models using AICM. Results show model fittings of Regional and Local phylogeographic model priors for the full DENV-1 genotype 1 data set and for 10 data sets including a maximum of 50 randomly subsampled sequences per location. Models with the best performance for each geographic and sampling scheme are indicated with a star.

To determine how viral transmission routes within Southeast Asia were influenced by population and geographic factors, we compared the fit of a variety of phylogeographic models related to human population size, distance, and DENV transmission intensities to the spatially- and temporally-related E gene sequence data. Although slightly different results were obtained for the Local and Regional models (Figure 3, Table 3), phylogeographic models that utilized simple distance- and population-based gravity model priors generally showed a good fit to the data relative to the equal rates and single factor models (distance, population), as indicated by lower values of marginal log likelihood AICM estimates. However, the incorporation of the Relative Endemicity factor (REf), which reflects DENV transmission intensities in both recipient and donor populations, further improved the fit of gravity models to the data under the Regional geographic scheme. The REf alone showed a consistently good fit to the data relative to other single factor models, and the best overall fit was shown by the REf+Gravity Model (Model 6) both for the full phylogeny and randomly subsampled data sets (50 per location). Importantly, the performance of the Sample Size model (Model 7) was not significantly better than other models of viral movement in the full or subsampled data sets. This indicates that spatial bias in sampling was not the most important factor determining the patterns of viral migration observed here.

**Table 3:** Marginal log likelihood estimates for the fit of different phylogeographic models using AICM.

	Local Model	Local Model subsamples (average)	Regional Model	Regional Model subsamples (average)
Equal Rates	1337.09	536.16	569.69	183.26
Distance	1335.20	539.13	575.51	174.38
Population	1345.07	559.98	579.98	179.17
Gravity Model	**1329.96**	**515.21**	563.65	172.92
REf	1336.76	524.41	567.91	173.00
REf+Gravity Model	1331.29	520.70	**560.06**	**165.30**
Sample Size	1331.86	558.84	570.29	178.20

Results show model fittings of Regional and Local phylogeographic model priors for the full DENV-1 genotype 1 data set and for 10 data sets including a maximum of 50 randomly subsampled sequences per location. Lower values of AIC indicate a better fit to the data. Models with the best performance for each geographic and data scheme are indicated in bold.

## Discussion

This study documents the dispersal of DENV-1 to populations across Viet Nam and provides evidence that viral populations are regularly introduced into northern Viet Nam from external populations but do not establish endemic cycles of transmission. Strong clustering at all spatial scales indicated that the viral diversity present in a given area is determined primarily by local gene flow. Frequent movement between neighboring locations under the Local geographic model may reflect a combination of human movement and vector dispersal in the movement of DENV between human populations in close proximity, as observed at smaller geographic scales [17], [18], [56]. However, many of the medium- and long-distance migration events observed here occur on a timescale of less than one year, suggesting that human-borne virus migration drives the long-range dispersal of DENV from south to north.

Our results also show that the DENV population in sub-tropical northern Viet Nam is characterized by regular seasonal invasions of lineages from the highly endemic south, with no detectable persistence into the following dengue season. Regardless of the time of year in which invasion occurred, our phylogenetic data suggest that invading lineages experience severe seasonal bottlenecks and regular fade-out in northern Viet Nam at the end of each year, when temperatures in much of the north drop below those considered optimal for survival of the vector and efficient transmission of the virus by *Aedes aegypti* [23]–[26]. However, the short-term transmission of strains of DENV in the first half of the year, which are suggested to have been introduced into the north during the cold, dry winter, indicate that these seasonal conditions are not sufficient to completely block transmission – perhaps due to residual, indoor breeding of *A. aegypti* mosquitoes. In contrast, strains of DENV introduced into central Viet Nam establish cycles of transmission extending over multiple years.

Clade 2 represents one possible exception to the observations above. Our analysis suggests that this lineage entered through the north and became established in the North Central Coast, where it may have persisted for over a decade prior to its invasion and establishment in the south. Although the possibility of long-term transmission in northern Viet Nam cannot be excluded, the lack of contemporaneous sequence data from the south and the distant relationships between these basal sequences result in a lack of resolution at this early time point and thus low support for an inferred ancestral location of the lineage. Additionally, a number of other sequences sampled from early time points in the north (Clade 4) also fall at basal positions in the phylogeny, although generally within the diversity of southern Viet Nam. Thus, the relationships among these northern sequences do not necessarily indicate their persistence. Instead, they may represent multiple importations from DENV populations in the south in the 1990s that experienced a significant bottleneck in the early 2000s, prior to the entry and rapid establishment of Clades 1, 4, 5, and 6. If the processes of DENV invasion in Viet Nam were similar to those observed at more recent time points when sampling was conducted across the country, we would expect that these northern sequences would fall into southern lineages that circulated prior to the bulk of our sampling. However, the lack of any signature of intermediate diversity suggesting the presence of this lineage in the highly sampled south prior to 2002 makes it difficult to test this hypothesis.

Among the more recently sampled sequences, there are a number of interesting patterns of viral dispersal within Viet Nam. Ho Chi Minh City and the Mekong Delta experience high transmission intensities and were highly sampled relative to the other populations (Table S1). Previous studies indicated that Ho Chi Minh City acted as a source of DENV diversity in the south [17], [18]. Here, we show that the role of the city as a primary source population extends across the entire country. The Mekong Delta, in contrast, was a source of DENV for populations only within the south. While fewer samples were available for the Southeast, this region appeared to be a significant source of viruses circulating in the north (Red River Delta and North Central Coast). The Southeast region has high population densities adjacent to HCMC and is the site of increasing numbers of industrial parks and emerging regional economic centers [57]. Economic migration to HCMC and the Southeast is especially common among young adults from the Mekong Delta, Central Coast regions, and the Red River Delta [58]. Importantly, young adults from the north may be dengue-naïve due to limited exposure to DENV, and may be at high risk of infection and illness after arriving in the southern industrial areas [59]. Movement of both migrants and short-term travelers between the economically important regions of Ho Chi Minh City, the Southeast, and Hanoi provides ample opportunity for the movement of DENV and a range of other important human pathogens. Improvements in road quality and accessibility to long-distance travel by air, land, and water over the last few decades have likely resulted in an increase in human movement throughout the country, and our results suggest that these movements may be partially responsible for changes in DENV activity in endemic and non-endemic regions. However, a lack of viral movement between the Southeast region and central Viet Nam, while similarly connected by human migration [58], may reflect (i) higher levels of immunity in the center of the country such that viral establishment is rare even when viral importation is frequent, (ii) differences in human movement between central Viet Nam and other regions, or (iii) insufficient sampling of this area. Indeed, a lower average age of dengue cases in this region and the finding that central Viet Nam maintains local lineages over multiple dengue seasons suggests that levels of population immunity here may be higher than in the north.

Previous studies in southern Viet Nam have suggested that viruses move through the area along somewhat predictable human migration routes based on estimates of physical and economic connectedness [17], [18]. Here, we compared a variety of epidemiological models reflecting patterns of human and mosquito movement on a much larger scale than the previous studies and showed that models incorporating patterns of human migration fit the data relatively well. The simple gravity model showed the best fit to the data under the Local geographic model and suggests that the movement of DENV between locations can be explained by the connectedness of the human populations at this scale. This is reflected in the frequency of medium- and long-distance viral migration events between population centers in our phylogenies. However, the addition of even a crude factor related to population immunity and transmission intensities (REf) in the recipient and donor populations improved the fit of the gravity model in the Regional geographic analysis. This discrepancy between the Local and Regional model is not surprising given that REf estimates were based on limited data and extrapolated to all locations within a region, and thus may not reflect complex heterogeneities in immunity and transmission at finer spatial scales. The finding that the REf performs well against other factors indicates that transmission intensities in both the recipient and donor populations play an important role in shaping the likelihood of viral invasion. Notably, however, the co-circulation and frequent invasion of DENV-1 lineages across all populations suggests that large susceptible populations exist across the region, even in areas of high transmission intensity. In this case, the role of immunity may be limited relative to the number of infections occurring in a given area over time. We acknowledge that the REf estimation is a greatly over-simplified indicator of relative population immunity and transmission intensities, and is based on extrapolation of the mean age of infection from multiple studies that used diverse methods and surveillance data sources. These data may not be directly comparable, and it is clear that this crude estimation technique and the underlying data should be refined.

Although we believe that REf calculations in part integrate issues related to vector biology, reflecting a lower force of infection in Singapore (where aggressive vector control efforts may limit transmission) and in northern Viet Nam (where winter temperatures likely limit vector density and competence), the lack of models that explicitly integrate vector biology is a weakness of this analysis. The availability of data on vector densities and species in the locations considered is limited, and the spatial and temporal scales of these analyses do not easily lend themselves to explicit consideration of vector dynamics in our models. As analytical methods and the scale of viral sampling improve, the use of phylogeographic methods that model the impact of factors such as vector density and vector competence on the dynamics of DENV in locations where such data are systematically collected may offer greater insight into the processes that mediate viral migration and establishment in new regions. Additionally, a better understanding of true human movements in the region (including air, land, and water routes) may allow us to further elucidate the relative roles of human movement, vector species and competence, and population immunity in the dispersal and persistence of DENV in these environments.

Phylogeographic inference may be strongly affected by uneven sampling in space and time [60]. Here, we employed multiple methods to control for both spatial and temporal biases in our data, and these consistently upheld most of the migration links between locations as inferred in our full DENV-1 data set. However, even under our subsampling schemes, the data were biased toward sequences from the south (2006–2008) and the small number of sequences obtained from areas in northern and central Viet Nam may have been insufficient to capture long-term persistence of rare lineages co-circulating with the dominant invading viruses. Additionally, differences in sampling over time hindered inference at deep locations in the tree and prevented us from determining the origins of Clade 2, a possible long-term northern DENV lineage. Importantly, the spatial sampling bias inherent to these data (85% of our Vietnamese sequences are from the south) reflects the reality of the dengue burden in the country, where 85% of all reported dengue cases occur in the south [20]. It is difficult to identify an appropriate sampling scheme for phylogeographic analysis, particularly given that large reductions in the number of samples from the south have the potential to greatly reduce the overall diversity of the data set. It remains to be determined whether more even sampling in all locations across time would yield different or more robust results, as this type of sampling could bias the analysis to represent an unrealistic epidemiological scenario. As studies of viral phylogeography become more common in diverse environments, it is important that appropriate methods of systematic sampling (and resampling) are developed to optimize inference under varied epidemiological and evolutionary scenarios.

Given recent increases in DENV incidence in northern Viet Nam and its geographic position at the margins of endemic transmission, additional sampling in this area is clearly warranted. Results here suggest that human populations that are connected to dengue-endemic regions may be at constant risk of DENV invasion if effective vector species are present, and that aggressive vector control measures may be necessary to prevent epidemics, even in sub-tropical and temperate regions with little to no history of DENV activity. Greater understanding of the processes by which DENV invades sub-tropical northern Viet Nam and the potential of this area to maintain long-term autochthonous viral transmission would yield important information relevant to sub-tropical and temperate areas at risk of DENV invasion worldwide.

## Supporting Information

Figure S1 **Inferred patterns of migration of DENV-1 across Viet Nam.** The number of arrows corresponds to the number of inferred migration events resulting in successful establishment of transmission in the recipient population with branch-specific Markov Jump probability ≥0.85.
(EPS)

Table S1 **Geographic distribution of DENV-1 sequences collected in Viet Nam from 1998 to 2009.** QUT indicates viruses collected and sequenced using the Queensland University of Technology protocol. OUCRU Hanoi and OUCRU HCMC indicate collected and sequenced using the protocols of the respective Oxford University Clinical Research Unit.
(DOCX)

Table S2 **Viral migration patterns in subsampled data sets.** The number of viral introductions is represented by Markov Jump counts (posterior expected number of state transitions between location in Southeast Asia, with 95% highest posterior density (HPD) intervals). All significant Markov Jump counts are shown.
(DOCX)
