# Supplementary material for: Dengue Virus in Sub-tropical Northern and Central Viet Nam: Population Immunity and Climate Shape Patterns of Viral Invasion and Maintenance
Source: PLoS Negl Trop Dis. 2013 Dec 5;7(12):e2581. doi: 10.1371/journal.pntd.0002581 (PMC3854975; doi:10.1371/journal.pntd.0002581)
Supplement: Table S1 — Geographic distribution of DENV-1 sequences collected in Viet Nam from 1998 to 2009. QUT indicates viruses collected and sequenced using the Queensland University of Technology protocol. OUCRU Hanoi and OUCRU HCMC indicate collected and sequenced using the protocols of the respective Oxford University Clinical Research Unit. (DOCX) [file pntd.0002581.s002.docx]

**Table S1. Geographic distribution of DENV-1 sequences collected in Viet Nam from 1998 to 2009.** QUT indicates viruses collected and sequenced using the Queensland University of Technology protocol. OUCRU Hanoi and OUCRU HCMC indicate collected and sequenced using the protocols of the respective Oxford University Clinical Research Unit.

| **Location** | **Number of Sequences** | **Year** | **Source** |
| --- | --- | --- | --- |
| **Red River Delta** | 1 | 2003 | QUT |
|  | 1 | 2004 | QUT |
|  | 23 | 2008 | 20 OUCRU Hanoi; 3 QUT |
|  | 5 | 2009 | QUT |
|  | *30* | *All* |  |
| **North Central Coast** | 1 | 1998 | QUT |
|  | 1 | 1999 | QUT |
|  | 3 | 2002 | QUT |
|  | 1 | 2003 | QUT |
|  | 4 | 2004 | QUT |
|  | 2 | 2006 | QUT |
|  | 1 | 2007 | QUT |
|  | 2 | 2008 | QUT |
|  | 1 | 2009 | QUT |
|  | *16* | *All* |  |
| **South Central Coast** | 1 | 2005 | QUT |
|  | 5 | 2006 | QUT |
|  | 7 | 2007 | 4 QUT; 3 OUCRU HCMC |
|  | 4 | 2008 | QUT |
|  | 12 | 2009 | QUT |
|  | *29* | *All* |  |
| **Central Highlands** | 5 | 2004 | QUT |
|  | *5* | *All* |  |
| **Southeast** | 6 | 2006 | OUCRU HCMC |
|  | 6 | 2007 | OUCRU HCMC |
|  | 14 | 2008 | OUCRU HCMC |
|  | *26* | *All* |  |
| **Ho Chi Minh City** | 9 | 2003 | OUCRU HCMC |
|  | 1 | 2004 | OUCRU HCMC |
|  | 13 | 2005 | OUCRU HCMC |
|  | 82 | 2006 | OUCRU HCMC |
|  | 87 | 2007 | OUCRU HCMC |
|  | 99 | 2008 | OUCRU HCMC |
|  | *291* | *All* |  |
| **Mekong Delta** | 1 | 2004 | OUCRU HCMC |
|  | 29 | 2006 | OUCRU HCMC |
|  | 86 | 2007 | OUCRU HCMC |
|  | 25 | 2008 | 24 OUCRU HCMC; 1 QUT |
|  | 3 | 2009 | QUT |
|  | *144* | *All* |  |
