# Supplementary material for: Dengue Virus in Sub-tropical Northern and Central Viet Nam: Population Immunity and Climate Shape Patterns of Viral Invasion and Maintenance
Source: PLoS Negl Trop Dis. 2013 Dec 5;7(12):e2581. doi: 10.1371/journal.pntd.0002581 (PMC3854975; doi:10.1371/journal.pntd.0002581)
Supplement: Table S2 — Viral migration patterns in subsampled data sets. The number of viral introductions is represented by Markov Jump counts (posterior expected number of state transitions between location in Southeast Asia, with 95% highest posterior density (HPD) intervals). All significant Markov Jump counts are shown. (DOCX) [file pntd.0002581.s003.docx]

**Table S2. Viral migration patterns in subsampled data sets.** The number of viral introductions is represented by Markov Jump counts (posterior expected number of state transitions between location in Southeast Asia, with 95% highest posterior density (HPD) intervals). All significant Markov Jump counts are shown.

| **Regional Model** | **TH to KH** | **SG to TH** | **SG to North VN** | **KH to South VN** | **South VN to Central VN** | **South VN to North VN** | | **Within South VN (Local Model Only)** | | | | | |
| --- | --- | --- | --- | --- | --- | --- | --- | --- | --- | --- | --- | --- | --- |
| **Subsample 5 per location per year (averaged)** | 4.2 (0.9, 6.1) | 2.1 (0.2, 4.2) | 2.5 (0.8, 4.6) | 3.5 (0.4, 6.8) | 11.5 (7.5, 14.9) | 9.8 (3.8, 16.2) | |  | | | |  |  |
| **Subsample 50 (averaged)** | 4.5 (1.3, 7.4) |  | 2.4 (0.9, 4.0) | 5.7 (2.1, 9.0) | 14.0 (10.3, 17.0) | 13.2 (8.6, 17.0) | |  | | | | | |
| **Local Model** | **TH to KH** |  |  | **KH to HCM** | **HCM to SCC** | **HCM to RRD** | **SE to RRD** | **HCM to SE** | **HCM to MKD** | **MKD to SE** | **MKD to HCM** | | |
| **Subsample 5 per location per year (averaged)** | 3.4 (0.1, 6.6) |  |  | 1.4 (0.1, 3.9) | 7.4 (1.9, 12.7) | 4.1 (0.1, 8.1) | 3.7 (0.4, 7.4) |  | 3.7 (0.1, 8.2) | 4.9 (1.2, 8.6) | 5.4 (0.6, 10.8) | | |
| **Subsample 50 (averaged)** | 3.6 (0.1, 6.7) |  |  | 2.1 (0.3, 5.0) | 11.2 (5.6, 16.0) | 4.8 (0.8, 8.7) | 9.3 (2.2, 11.1) | 7.7 (0.9, 13.7) | 10.2 (2.5, 17.5) | 15.0 (8.7, 21.0) | 13.9 (5.1, 22.4) | | |
